# Dual effects of serum urate on stroke risk and prognosis: insights from Mendelian randomization

**DOI:** 10.3389/fneur.2024.1359292

**Published:** 2024-04-02

**Authors:** Shixuan Chen, Zhenzhen Chen, Qingqing Xu, Xia Jiang, Chuyong Lin, Jing Ji

**Affiliations:** ^1^Department of Rehabilitation Medicine, Wenzhou Hospital of Integrated Traditional Chinese and Western Medicine, Zhejiang Chinese Medical University, Wenzhou, China; ^2^Department of Nursing, Wenzhou Hospital of Integrated Traditional Chinese and Western Medicine, Zhejiang Chinese Medical University, Wenzhou, China

**Keywords:** uric acid, stroke, blood pressure, genetic instrumental variables, Mendelian randomization

## Abstract

**Background:**

To investigate the causal associations of serum urate (SUA) with stroke risk and prognosis using Mendelian randomization (MR) and the potential mediating role of stroke risk factors in the causal pathways.

**Methods:**

We used the random-effects inverse variance weighting (IVW) as our primary method. We initially performed two-sample univariable MR (UVMR) to identify the causal associations of SUA (*n* = 437,354) with any stroke (AS, FinnGen: *n* = 311,635; MEGASTROKE: *n* = 446,696), ischemic stroke (IS, FinnGen: *n* = 212,774; MEGASTROKE: *n* = 440,328), intracranial hemorrhage (ICH, FinnGen: *n* = 343,663; ISGC: *n* = 3,026), functional outcome after ischemic stroke at 90d (*n* = 4,363), and motor recovery within 24 months after stroke (*n* = 488), and then multivariable MR (MVMR) to estimate the direct causal effects of SUA on these outcomes, adjusting for potential confounders. Finally, we further conducted a two-step MR to investigate the potential mediating role of body mass index (BMI), systolic blood pressure (SBP), diastolic blood pressure (DBP), and estimated glomerular filtration rate (eGFR) in the identified causal pathways.

**Results:**

Genetically predicted elevated SUA levels were significantly associated with increased risk of AS (meta-analysis: OR = 1.09, 95% CI [1.04–1.13], *p* = 3.69e-05) and IS (meta-analysis: OR = 1.10, 95% CI [1.01–1.19], *p* = 0.021) and with improved poor functional outcome after ischemic stroke at 90d (OR = 0.81, 95% CI [0.72–0.90], *p* = 1.79e-04) and motor recovery within 24 months after stroke (OR = 1.42, 95% CI [1.23–1.64], *p* = 2.15e-06). In MVMR, SBP and DBP significantly attenuated the causal effects of SUA on AS, IS, and functional outcome after ischemic stroke at 90d and motor recovery within 24 months after stroke. Further mediation analyses showed that SBP mediated 52.4 and 34.5% of the effects of SUA on AS and IS, while DBP mediated 28.5 and 23.4% of the causal effects, respectively.

**Conclusion:**

This study supports the dual role of genetically predicted SUA in increasing stroke risk, especially ischemic stroke risk, and in improving functional outcome and motor recovery. SBP and DBP are key mediators lying on the causal pathways of SUA with AS and IS.

## 1 Introduction

Stroke is the major cause of mortality and disability worldwide, imposing a substantial challenge to human health. Despite considerable efforts dedicated to stroke prevention and treatment, its prevalence and disability rates remain high, signaling ongoing gaps in understanding and management strategies for stroke. Serum urate (SUA), the final output of purine metabolism, possesses pro-oxidant and antioxidant dual properties and may exert complex biological effects in our body. Observational studies showed that high SUA levels may indirectly contribute to stroke by inducing inflammatory responses, promoting oxidative stress, and triggering endothelial dysfunction (1–3). Additionally, SUA is also implicated in increasing thrombosis risk, which may impact the occurrence of cardiovascular disease (4). However, some studies have argued that the link between SUA and stroke is still a “pseudo-association” as hyperuricemia is closely associated with other stroke risk factors such as hypertension and obesity; thus, whether or not SUA affects stroke is controversial (5, 6). Furthermore, interventions aimed at reducing SUA did not prevent the onset and progression of cardiovascular disease, which further raises doubts about a direct link between SUA and stroke (7). On the other hand, there are conflicting findings regarding the influence of SUA on stroke prognosis (8–10). Therefore, it is necessary to conduct a more in-depth investigation into the causal association of SUA with stroke risk and prognosis as well as to unravel their precise mechanisms.

Conventional epidemiologic studies are susceptible to unaccounted confounders, excessive adjustment for mediators, or reverse causality, potentially leading to bias in the established causal associations. Mendelian randomization (MR) is an emerging approach for causal inference assessment that cleverly exploits the random distribution of genetic variants as instrumental variables (IVs) at conception to simulate the “random assignment” of participants in randomized controlled trials (RCTs) and aims to identify the differential risk of disease between genetic variant carriers and non-carriers (11). Due to genetic variation being inherited at birth and remaining stable throughout our lifespan, associations obtained from MR are less susceptible to causal inversions and unaccounted confounders compared to traditional observational studies. This study employed a two-sample MR to identify causal associations of SUA with stroke, post-stroke functional outcome, and motor recovery. Additionally, we further performed a two-step MR to explore the potential mediating roles of mediators in the identified causal pathways, which may contribute to enhancing our understanding of the mechanisms linking SUA to stroke risk and prognosis.

## 2 Methods

### 2.1 Study design

**Figure 1:**
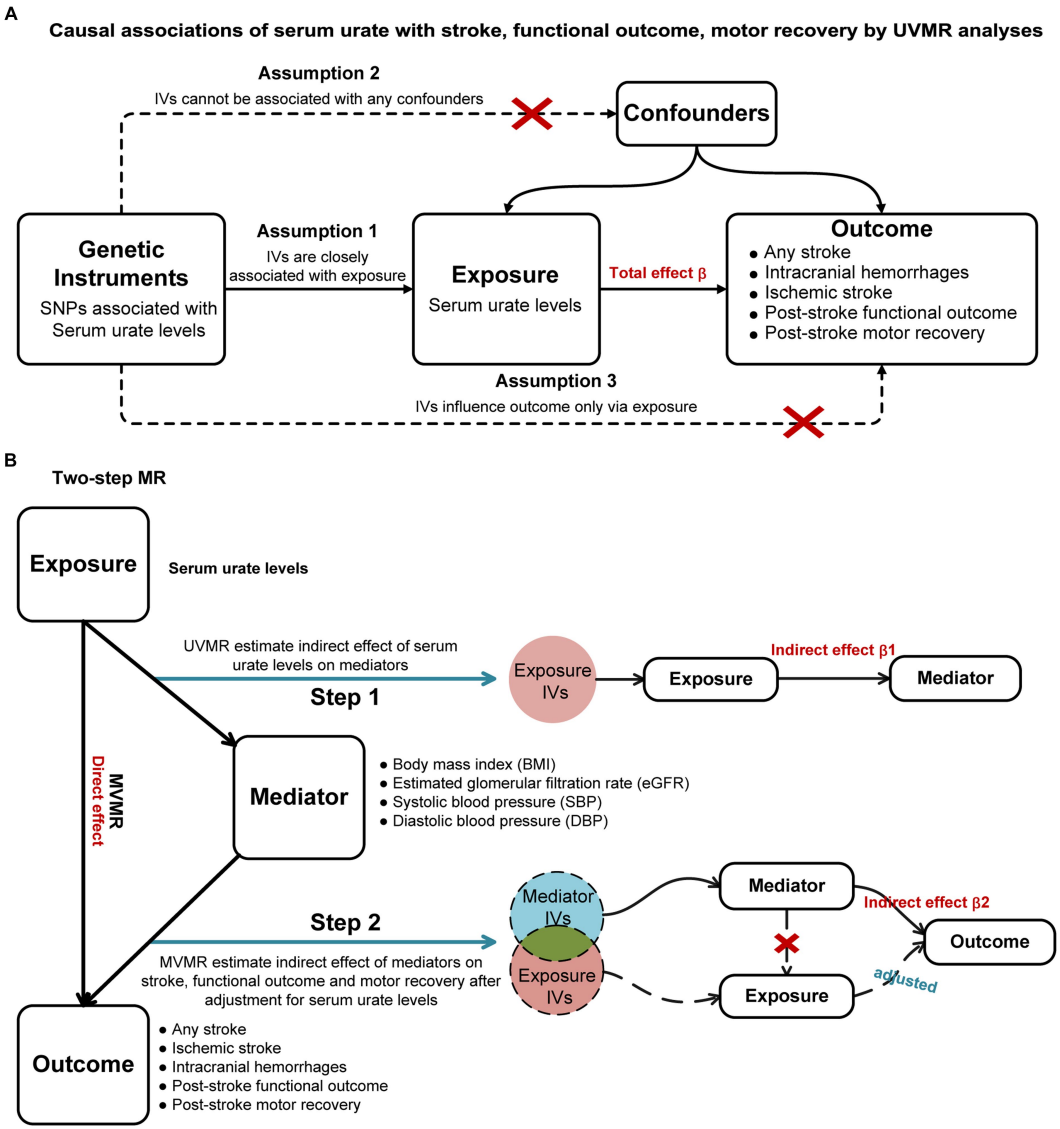
Study workflow overview. **(A)**: Causal associations of serum urate with stroke, functional outcome, motor recovery by UVMR analyses; **(B)**: Two-step MR. UVMR, univariable Mendelian randomization; MVMR, multivariable Mendelian randomization; IVs, instrumental variables.

The workflow is visually presented in Figure 1. We initially performed a two-sample univariable MR (UVMR) to identify potential causal associations of SUA levels with stroke, post-stroke functional outcome, and motor recovery. A multivariable MR (MVMR) was then utilized to estimate the direct causal effects of SUA on these outcomes, adjusting for potential confounders. Moreover, we conducted a two-step MR to investigate the potential mediating role of body mass index (BMI), systolic blood pressure (SBP), diastolic blood pressure (DBP), and estimated glomerular filtration rate (eGFR) in the identified causal pathway. This MR study is based on publicly available published GWAS summary statistics, and all necessary ethical approval and informed consent were obtained for the original study.

### 2.2 Genetic instrument selection

SUA was included as exposure in this study, with its genome-wide association study (GWAS) dataset obtained from Barton AR et al.’s further analysis of the UK Biobank data involving 437,354 individuals of European ancestry (12). The SUA levels were measured by uricase PAP analysis, and the processed data were expressed as per standard deviation (1-SD = 80.3 μmol/L) of increase. We selected significant (*p* < 5 × 10^−8^) and independent (*r*^2^ < 0.001 and a distance window of 10,000 kb) single nucleotide polymorphisms (SNPs) as genetic IVs for SUA. Subsequently, SNPs in palindromes and those that were closely linked to outcome (*p* < 5 × 10^−5^) were excluded. We further filtered out the SNPs with the MR Steiger test, which primarily affected the outcomes, rather than the exposures. Moreover, we calculated F-statistics to evaluate the potential bias caused by weak instruments using the following formula: $F=\left(N-k-1\right)/k\times R^{2}∕\left(1-R^{2}\right)$ , where N , k , and $R^{2}$ are sample size, number of IVs, and variance explained by all IVs, respectively (13). $R^{2}=2\times EAF\times \left(1-EAF\right)\times \mathrm{bet}a^{2}$ , where EAF are effect allele frequency and beta are exposure effect. Any IV with an *F*-value below 10 suggests weakness and was excluded (13). Proxy SNPs were not available for our analysis. All genetic IVs used to proxy for SUA are listed in Supplementary Table S2.

### 2.3 Outcome data sources

Our outcomes included any stroke (AS), ischemic stroke (IS), intracranial hemorrhage (ICH), post-stroke functional outcome, and motor recovery. GWAS data for AS (39,818 cases, 271,817 controls), IS (10,551 cases, 202,223 controls), and ICH (3,749 cases, 339,914 controls) were obtained from the FinnGen Consortium (14). AS included any stroke, mainly IS, transient ischemic attack (TIA), hemorrhagic stroke, and subarachnoid hemorrhage. IS referred to any ischemic stroke excluding all hemorrhages, and ICH specifically denoted all cases of hemorrhage excluding IS and TIA. These diseases were principally defined based on ICD diagnosis codes at the time of discharge or death. We additionally obtained GWAS for AS (40,585 cases and 406,111 controls) and IS (34,217 cases and 406,111 controls) from the MEGASTROKE consortium, and ICH (1,545 cases and 1,481 controls) from the ISGC consortium (15, 16). GWAS summary data for functional outcome after ischemic stroke at 90d were derived from the Genetics of Ischemic Stroke Functional Outcome (GISCOME) network, which included 4,363 individuals of European ancestry (17). Functional outcome was assessed using the modified Rankin Scale (mRS) approximately 90 days after the stroke. The mRS score of 0–1 is defined as a good functional outcome (1,796 cases) and scores of 2–6 are considered poor functional outcomes (2,567 cases). Functional outcomes, adjusted for age, sex, ancestry, and baseline stroke severity as assessed by the National Institutes of Health Stroke Scale (NIHSS), were used for discovery analyses, while those unadjusted for baseline NIHSS were utilized for validation analyses. GWAS data for post-stroke motor recovery were obtained from further analysis of the Vitamin Intervention for Stroke Prevention (VISP) dataset by Aldridge CM et al., including 488 European individuals (18). This study applied the NIHSS subscores 5A/5B and 6A/6B to assess motor drift scores of limb motor weakness at six time points over 24 months. A drift score decrease of ≥1 vs. < 1 at each time point was taken as the study outcome. Detailed information on outcomes is presented in Supplementary Table S1.

### 2.4 Statistical analysis

We initially performed UVMR analyses, and the multiplicative random-effects inverse variance weighting (IVW) was adopted as our primary method to identify the causal associations of SUA on stroke, post-stroke functional outcome, and motor recovery, respectively. In the assumption that all IVs were valid, the IVW could provide the most robust estimate for MR (19). Complementary analyses to examine the consistency of the results included MR-Egger, weighted median, and MR pleiotropy residual sum and outlier (MR-PRESSO). MR-Egger regression incorporated an intercept term, which still yielded a reliable causal estimate even if all the IVs were invalid. Additionally, the weighted median method could provide a consistent estimate when the assumption of over 50% IVs validity was fulfilled. Cochran’s *Q* statistical tests were utilized to detect heterogeneity in estimates among SNPs, while the intercept derived from MR-Egger assessed horizontal pleiotropy. A significance level of *p* < 0.050 indicated the presence of heterogeneity or horizontal pleiotropy. Moreover, we performed the MR-PRESSO test to detect and correct horizontal pleiotropy outliers by removing them. To control for false-positive results due to multiple testing, the *p*-value for Bonferroni-corrected IVW was set at *p* < 0.006 (0.050/9 outcomes). The nominally significant *p*-value was defined as 0.006 ≤ *p* < 0.050, indicating suggestive evidence for potential causality. We further utilized the online tool^1^ to estimate the statistical power for various outcomes. A higher power value indicates a greater certainty in detecting significant effects.

For significant causality in UVMR, we applied the random-effects IVW model within the MVMR analysis. This approach adjusted for potential confounders such as BMI, eGFR, SBP, and DBP, enabling us to estimate a direct causal effect. To further ensure the reliability of our findings, we conducted sensitivity analyses using MR-Egger and weighted median methods. These analyses were particularly important due to the close metabolic relationship between hyperuricemia, BMI, and eGFR, as well as the fact that elevated SUA levels independently contribute to high blood pressure, a significant risk factor for cardiovascular disease. To explore the potential effects of mediators in this causal pathway, we further performed a two-step MR analysis. In step 1, UVMR was performed to estimate the indirect effects of SUA on the mediators (β1), and in step 2, we conducted MVMR to estimate the indirect effects of mediators on stroke, post-stroke functional outcome, and motor recovery after adjusting for SUA (β2). Finally, we estimated the significance of mediating effects (β1*β2) using the delta method, and then, their proportion in the total effect was calculated as proportion% = (β1*β2)/β*100%, where β was the total causal effect of SUA on stroke, post-stroke functional outcome, or motor recovery.

All analyses were conducted by using the “TwoSampleMR (version 0.5.6),” “MR-PRESSO (version 1.0),” “MVMR,” and “MendelianRandomization (version 0.8.0)” packages in R (version 4.3.1; The R Foundation for Statistical Computing).

## 3 Results

### 3.1 Univariable MR analysis

**Figure 2:**
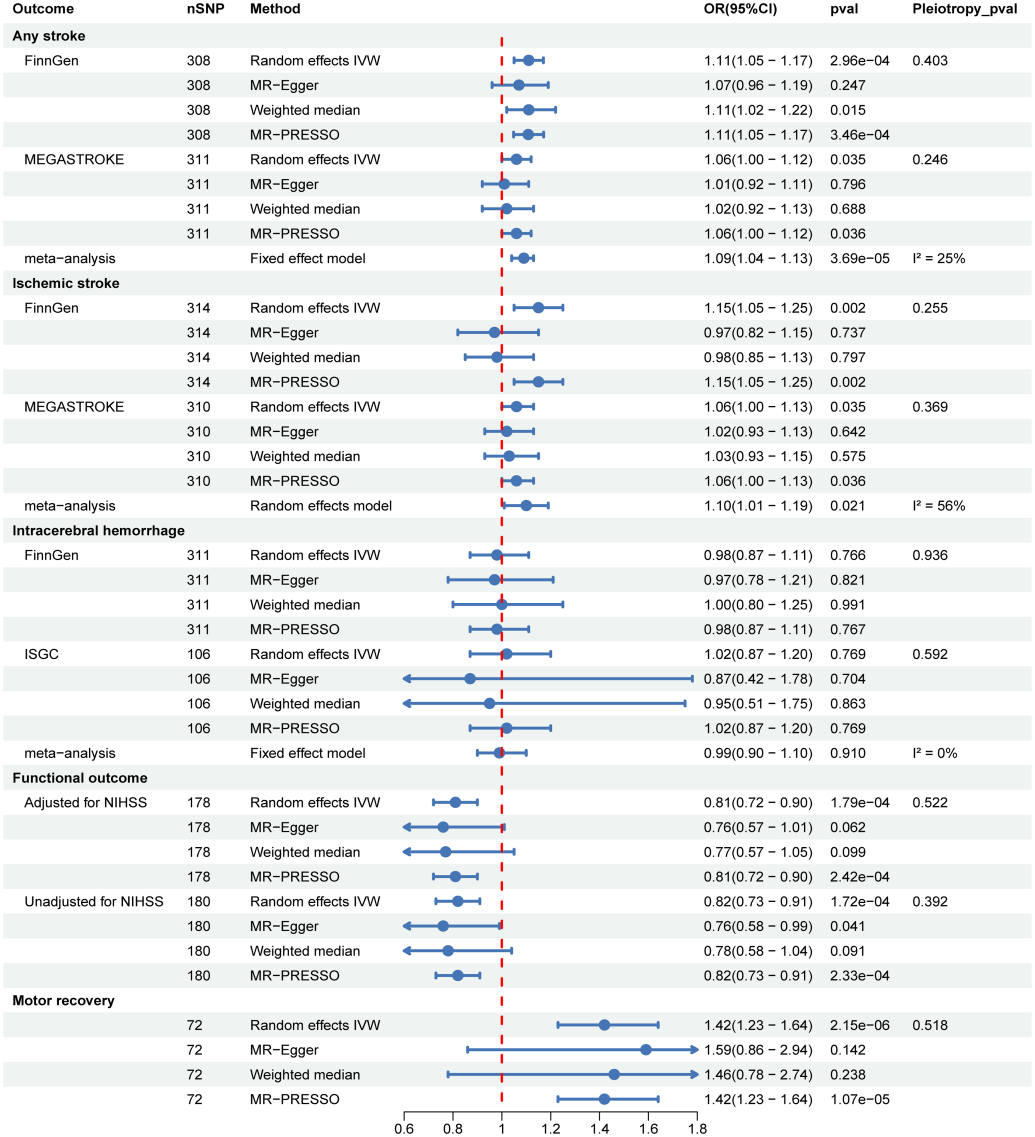
Two-sample univariable MR to identify the causal associations of SUA with stroke, post-stroke functional outcome, and motor recovery.

We performed UVMR to identify the causal associations between genetically predicted SUA and stroke, functional outcome after ischemic stroke at 90d, and motor recovery within 24 months after stroke, as well as to estimate their total causal effects. As shown in Figure 2 and Supplementary Table S3, the primary random-effects IVW in the UVMR analysis showed credible evidence that genetically predicted higher SUA levels were associated with increased risk of AS and IS, the results from the FinnGen and MEGASTROKE consortiums were highly consistent, and further meta-analysis of their IVW results indicated that each 1-SD increase in genetically predicted SUA was associated with a 9% higher risk of developing AS (OR = 1.09, 95% CI = 1.04–1.13, *p* = 3.69e-05; *I*^2^ = 25%, *p*_heterogeneity_ = 0.250) and IS (OR = 1.10, 95% CI = 1.01–1.19, *p* = 0.021; *I*^2^ = 56%, *p*_heterogeneity_ = 0.130). However, no significant causal association between SUA and ICH was shown in the FinnGen and ISGC consortiums, nor their meta-analysis (OR = 0.99, 95% CI = 0.90–1.10, *p* = 0.910; *I*^2^ = 0%, *p*_heterogeneity_ = 0.700). Significantly, we found that higher SUA levels were negatively linked to poor functional outcome after ischemic stroke at 90d, with each 1-SD increase in genetically predicted SUA levels being linked to a 19% lower risk of poor functional outcome (OR = 0.81, 95% CI = 0.72–0.90, *p* = 1.79e-04), and this result was consistent with the unadjusted NIHSS data (OR = 0.82, 95% CI = 0.73–0.91, *p* = 1.72e-04). Additionally, our results also indicated a positive correlation between higher SUA levels and motor recovery within 24 months after stroke, with each 1-SD increase in genetically predicted SUA levels associated with a 42% improvement in motor recovery (OR = 1.42, 95% CI = 1.23–1.64, *p* = 2.15e-06).

Sensitivity analysis methods, including MR-Egger, weighted median, and MR-PRESSO, were conducted to assess the robustness of the results. Although some of the sensitivity analysis methods did not yield significance estimates consistent with IVW, particularly MR-Egger, we noted the direction of causal estimates for the majority of them that were consistent with IVW (Figure 2 and Supplementary Table S3). Notably, no evidence of horizontal pleiotropy was found in any of the MR-Egger intercept tests, all of which were above 0.05 (Figure 2 and Table 1). In addition, we further performed MR-PRESSO analysis and found some outliers (Supplementary Table S3). Following excluding these outliers, UVMR analyses were repeated to obtain the final estimates. Finally, we performed reverse UVMR analyses, wherein no evidence of bidirectional causal associations of SUA levels with AS, IS, ICH, poststroke functional outcomes, or motor recovery were detected (Supplementary Table S4).

**Table 1:** Heterogeneity and horizontal pleiotropy in the causal associations of SUA with stroke, post-stroke functional outcome, and motor recovery as detected using IVW and MR-Egger.

Outcome	nSNP	Heterogeneity (IVW)		Pleiotropy (MR-Egger)		*R*^2^ sums	*F*-statistic mean	Power (%)
		Cochran’s Q	*p*-value	Intercept	*p*-value			
Finngen_AS	308	432.378	3.01E-06	0.001	0.403	0.063	89.323	99.8
MEGASTROKE_AS	311	364.981	0.017	0.001	0.246	0.072	101.626	83.5
Finngen_IS	314	351.563	0.066	0.005	0.255	0.065	89.940	95.6
MEGASTROKE_IS	310	357.255	0.030	0.001	0.369	0.072	101.838	83.5
Finngen_ICH	311	289.661	0.791	0.000	0.936	0.073	102.223	4.8
ISGC_ICH	106	17.331	1.000	0.006	0.592	0.032	131.441	3.3
Functional outcome adjusted NIHSS	178	45.992	1.000	0.004	0.522	0.105	263.976	62.0
Functional outcome unadjusted NIHSS	180	49.019	1.000	0.005	0.392	0.105	262.607	56.5
Motor recovery	72	5.836	1.000	−0.012	0.518	0.087	541.893	20.4

SUA, serum urate; Finngen_AS, Finngen for any stroke; MEGASTROKE_AS, MEGASTROKE for any stroke; Finngen_IS, Finngen for ischemic stroke; MEGASTROKE _IS, MEGASTROKE for ischemic stroke; Finngen_ICH, Finngen for intracranial hemorrhage; ISGC_ICH, ISGC for intracranial hemorrhage; NIHSS, National Institute of Health Stroke Scale.

### 3.2 Multivariable MR analysis

**Figure 3:**
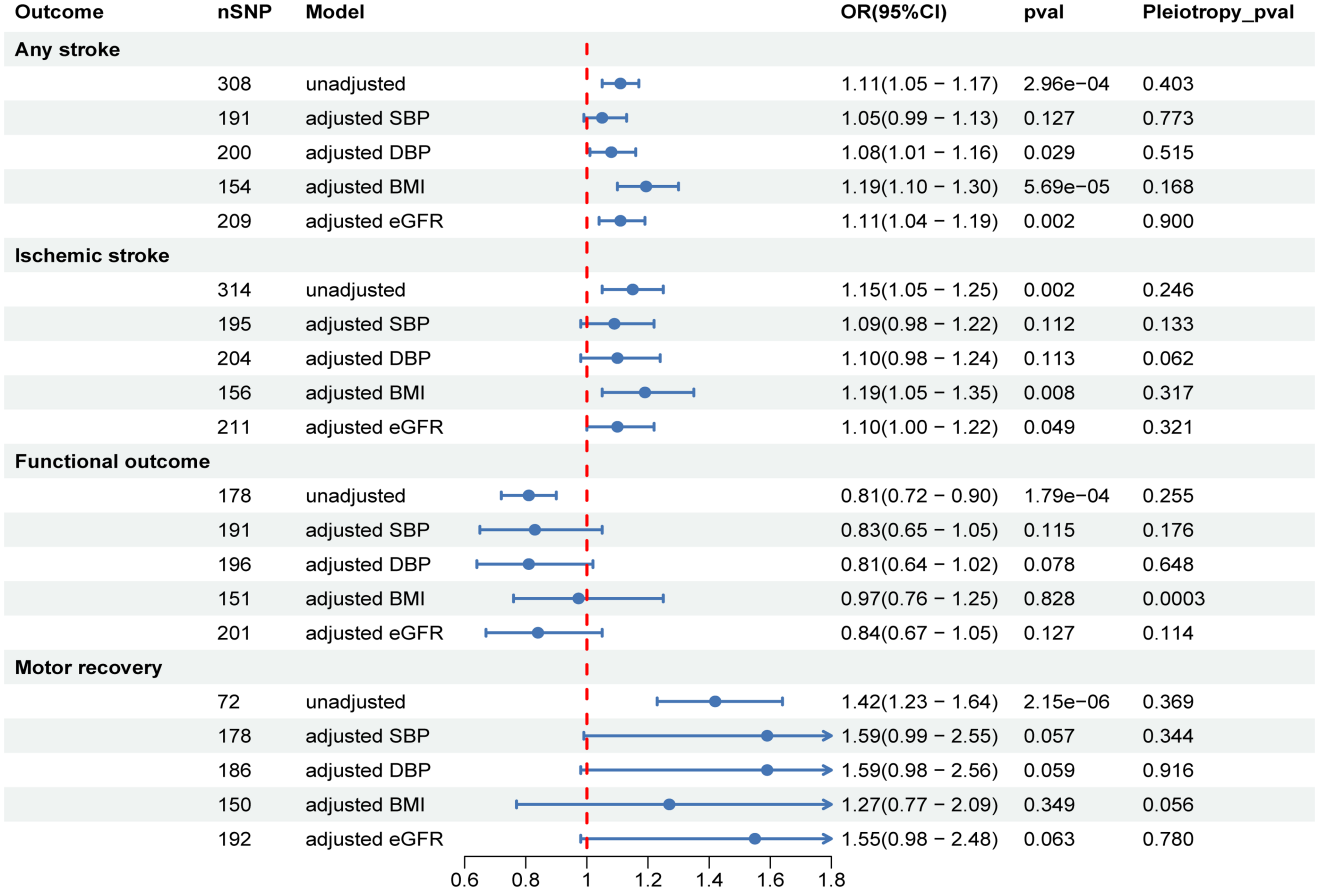
Multivariable MR to analyze the causal effects of SUA on stroke, post-stroke functional outcome, and motor recovery after adjusting for confounders and to estimate their direct effects. SBP, systolic blood pressure; DBP, diastolic blood pressure; BMI, body mass index; eGFR, estimated glomerular filtration rate.

After adjusting for BMI and eGFR using MVMR, the causal effect of SUA on AS and IS remained significant, but the estimates were attenuated or no longer significant after adjusting for SBP and DBP. Furthermore, after adjusting for BMI, eGFR, SBP, and DBP, the causal effect of SUA on functional outcome and motor recovery was no longer significant (Figure 3 and Supplementary Table S5).

### 3.3 Mediation analysis

**Figure 4:**
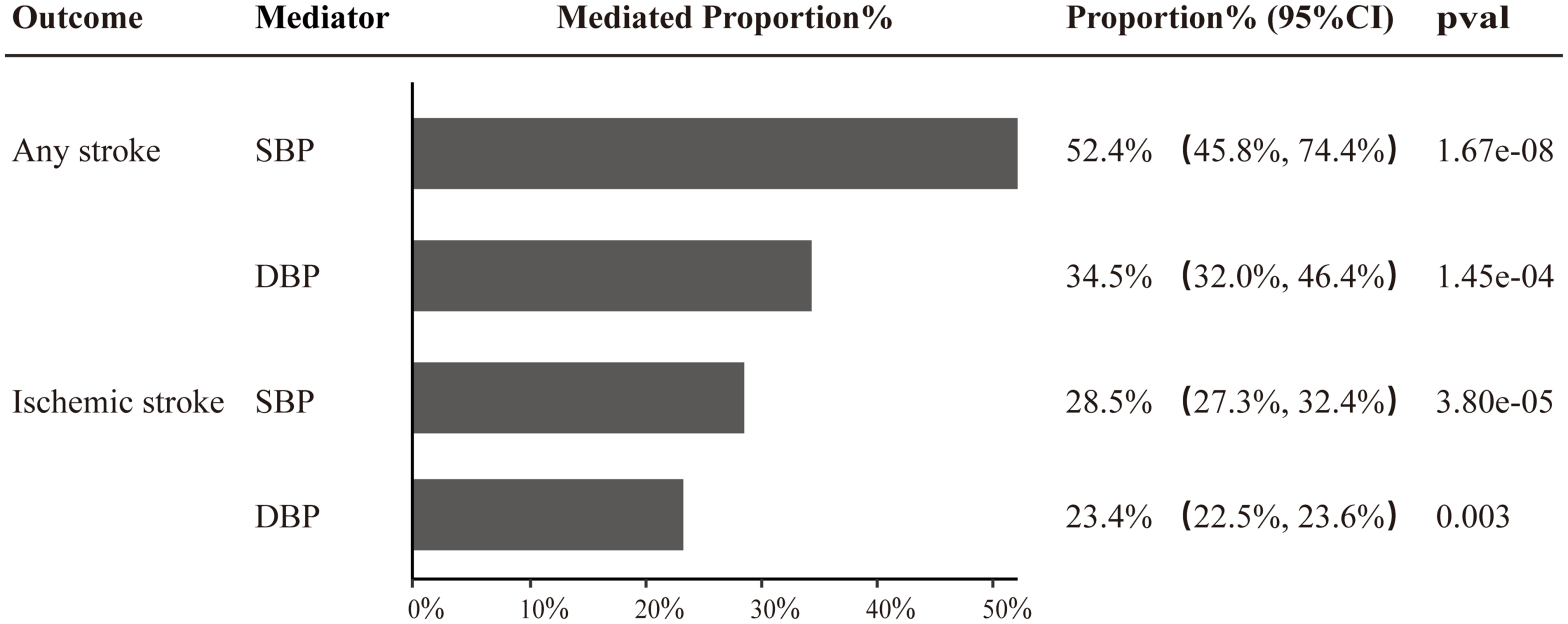
Two-step MR estimates of causal effect proportions mediated by SBP and DBP in SUA on any stroke and ischemic stroke pathways.

Two-step MR was utilized to conduct mediation analyses aimed at investigating whether the identified causal effect of SUA on stroke (FinnGen data), post-stroke functional outcomes (adjusted for NIHSS), and motor recovery could be mediated by SBP, DBP, BMI, or eGFR. Of note, our study revealed that SBP and DBP exerted proportionally mediating effects on the causal associations of SUA with AS and IS. Specifically, SBP was found to mediate 52.4% (95% CI: 45.8–74.4%, *p* = 1.67e-08) and 34.5% (95% CI: 32.0–46.4%, *p* = 1.45e-04) of the causal effects of SUA on AS and IS, respectively, while DBP mediated 28.5% (95%CI: 27.3–32.4%, *p* = 3.80e-05) and 23.4% (95%CI: 22.5–23.6%, *p* = 0.003) of the causal effects on AS and IS, respectively (Figure 4 and Supplementary Table S6).

## 4 Discussion

To investigate the causal associations and mechanisms of SUA on stroke, post-stroke functional outcome, and motor recovery, we performed this MR study utilizing large-scale, publicly available GWAS summary data. Four critical findings emerged from our analysis: (1) Genetically predicted elevated SUA levels were significantly causally associated with an increased risk of AS and IS, and these associations remained significant even after adjusting for BMI and eGFR. (2) SBP and DBP were identified as critical mediators in the causal pathways linking SUA to AS and IS. (3) Elevated SUA levels were found to lower poor functional outcomes after 90d of ischemic stroke and also contributed to motor recovery within 24 months after stroke. (4) There was no evidence of a significant causal association between SUA and ICH. Additionally, sensitivity analyses suggested consistent estimates and effect directions for almost all outcomes, and no horizontal pleiotropy was found.

Previous observational studies have explored the link between SUA and stroke but yielded conflicting conclusions (6, 20, 21). Epidemiologic studies often struggle to fully exclude confounders and address reverse causality, which may be key drivers of inconsistent conclusions. MR using genetic variants as IVs to proxy for different phenotypes contributes to maximizing control for confounders and avoids reverse causality interference, thus providing more reliable causal inferences. A recent MR study suggested that higher SUA levels would increase AS risk, with each 1-SD (80.3 μmol/L) increase in genetically predicted SUA levels being associated with an 11% increase in the risk of developing AS (22). Our MR analyses using GWAS data from different sources yielded similar results. The subsequent meta-analysis of the IVW estimates derived from these analyses obtained a more significant OR estimate and a narrower 95% confidence interval. IS is the primary subtype of AS, and evidence from two previous MR studies involving SUA and IS did not support a causal association among them (23, 24). These were contradictory to our findings. Given that our MR extracted more eligible IVs (310–314 vs. 28 SNPs) and employed GWAS summary data only from European ancestry, this enhances the reliability of our findings. Additionally, the study further indicated that there was no causal association between SUA and ICH. Therefore, a previous observational study reported that a positive relationship of stable high SUA with ICH risk but not with IS may have been coincidental (21).

Currently, there is a lack of consensus on the exact role of SUA in stroke prognosis (25, 26). In a recent MR study, SUA was not found to be causally associated with poor functional prognosis (mRS > 2) at 90d after ischemic stroke (27). However, our MR analysis, using GWAS data defining mRS > 1 as poor functional outcome, identified a significant and negative causal association between SUA and poor functional prognosis in ischemic stroke patients at 90d. Prior meta-analysis suggested a non-linear (U-shaped) association between SUA and the risk of poor functional outcomes after ischemic stroke (28). This complex relationship between SUA and post-stroke functional prognosis may be the key factor contributing to this contradictory result. Additionally, our MR analyses found a positive causal association between SUA and motor recovery within 24 months after stroke. This is consistent with previous findings that SUA exerted antioxidant properties and potential neuroprotective effects (29). However, further studies are still required to fully grasp the complexity and potential non-linear relationship between SUA and post-stroke prognosis.

Through this study, we have found that SUA has dual effects, with elevated SUA being a risk factor for AS and IS, while also exerting a protective effect on post-stroke functional outcome and motor recovery. However, the exact mechanism between them remains ambiguous. Numerous studies suggest that hyperuricemia plays an important role in the etiology of hypertension, which is the leading risk factor for stroke (30, 31). Our further mediation analyses identified SBP and DBP as key mediators lying on the SUA to AS and IS are causal pathways, which is consistent with the result previously reported by Chaudhary NS et al. (31). Additionally, blood pressure is the primary driver of maintaining cerebral perfusion. Sustaining appropriate blood pressure in the early stages of ischemic stroke is beneficial for cerebral perfusion and aids in neurological function recovery. These factors, in turn, positively influence stroke prognosis (32). Of note, although we failed to detect the mediating effect of blood pressure in the pathway of SUA on stroke prognosis, the influence of SUA on post-stroke functional outcome and motor recovery became non-significant after adjusting for SBP and DBP using MVMR. This evidence provided insights into the role of SUA in the pathogenesis and rehabilitation of stroke and may benefit future research and clinical practice.

Overweight is also a well-known risk factor for stroke. Our analyses revealed that BMI did not act as a mediator or attenuate the impact of SUA on AS and IS, suggesting that SUA increases the stroke risk and may be independent of BMI. It is important to note that being overweight is not always detrimental. Some studies have suggested that being overweight indicated the presence of more nutritional reserves, which could potentially help counteract post-stroke hypermetabolic depletion, thus translating into a protective factor against poor functional outcomes (33, 34). This might be a potential explanation for our finding that the causal effect of SUA on post-stroke functional outcome and motor recovery became non-significant after adjusting for BMI. Additionally, impaired kidney function is an independent risk factor for stroke and is linked to more severe stroke and worse outcomes (35). In our analysis, we also found that eGFR attenuated the causal effect and significance of SUA on IS. Although further mediation analysis failed to yield a significant mediating effect, it still implied that the improvement of post-stroke functional outcome and the promotion of motor recovery by SUA may rely on favorable renal function.

Our study holds several strengths as follows. First, the study employed MR to investigate the causal association between SUA and stroke risk and prognosis, which minimized bias from confounders and reverse causality, thus providing credible causal inferences. Second, our efforts identified the dual effects of SUA on stroke risk and prognosis, and a series of replication analyses, GWAS meta-analyses, and sensitivity analyses observed similar results, ensuring the robustness of the findings. Finally, potential mechanisms of stroke risk factors in causal pathways were explored by MVMR and mediation analyses, and this insight may guide the development of targeted intervention and prevention strategies.

The study also has some limitations. First, the potential pleiotropy is an inherent limitation of MR analysis. Despite performing strict selection criteria for genetic IVs and examining for outliers and horizontal pleiotropy, pleiotropy may still be present and could introduce bias into our results. Second, it is necessary to point out the genetic variation differences that exist among different races, which may introduce heterogeneity into the causal estimates. Although the incidence of stroke is also high in the Asian region (36–38), considering that our study mainly involves participants of European descent, this may limit the generalization of our findings to other races and populations. Third, in this study, we noted a 43.6% sample overlap in SUA and eGFR GWAS datasets, potentially impacting the accuracy of MVMR and mediation analysis. Due to the absence of independent GWAS datasets excluding UK Biobank, we evaluated the robustness of our findings by estimating potential bias and type I error rate. The finding indicates that a 43.6% overlap might cause a bias ranging from 0.007 to 0.008 and increase type I error rates between 0.19 and 0.27. Although the bias suggests a somewhat reliable result, the elevated type I error rates, substantially above the 5% standard threshold, indicate a heightened risk of false positives. Future studies with non-overlapping samples are required to corroborate and reinforce our conclusions. Finally, limited by the GWAS data, we were unable to stratify SUA levels or explore trend relationships between SUA and stroke risk and prognosis. In future studies, a more comprehensive collection of SUA data and more in-depth analysis will contribute to further insight into the potentially complex associations between them.

In conclusion, our MR study supports a dual role for genetically predicted SUA in increasing the risk of stroke, especially ischemic stroke, and in improving post-stroke poor functional outcome and motor recovery. Moreover, we provide credible genetic evidence that SBP and DBP mediate considerable proportions of the causal effects of SUA on AS and IS. These findings contribute to a deeper understanding of the underlying mechanisms that link SUA to stroke risk and prognosis.

## Data availability statement

The original contributions presented in the study are included in the article/Supplementary material, further inquiries can be directed to the corresponding author.

## Ethics statement

Ethical review and approval was not required for the study on human participants in accordance with the local legislation and institutional requirements. Written informed consent for participation was not required for this study in accordance with the national legislation and the institutional requirements.

## Author contributions

SC: Conceptualization, Funding acquisition, Methodology, Writing – original draft. ZC: Data curation, Visualization, Writing – original draft. QX: Data curation, Writing – original draft. XJ: Formal analysis, Validation, Writing – original draft. CL: Formal analysis, Writing – original draft. JJ: Conceptualization, Funding acquisition, Methodology, Writing – original draft.

## Footnotes

^1^ https://sb452.shinyapps.io/power
